# Different angiotensin receptor blockers and incidence of diabetes: a nationwide population-based cohort study

**DOI:** 10.1186/1475-2840-13-91

**Published:** 2014-05-14

**Authors:** Chia-Hsuin Chang, Yi-Cheng Chang, Li-Chiu Wu, Jou-Wei Lin, Lee-Ming Chuang, Mei-Shu Lai

**Affiliations:** 1 Institute of Preventive Medicine, College of Public Health, National Taiwan University, Taipei, Taiwan; 2 Department of Internal Medicine, National Taiwan University Hospital, 7, Chung Shan S. Rd, Taipei, Taiwan; 3 Department of Medicine, College of Medicine, National Taiwan University, Taipei, Taiwan; 4 Department of Internal Medicine, National Taiwan University Hospital HsinChu Branch, HsinChu, Taiwan; 5 Cardiovascular Center, National Taiwan University Hospital Yun-Lin Branch, 579 Yun-Lin Road, Section 2, Dou-Liou City, Yun-Lin County, Taiwan

**Keywords:** Angiotensin receptor antagonists, Diabetes mellitus, Cohort studies

## Abstract

**Background:**

Angiotensin receptor blockers (ARBs) have been shown to exert various peroxisome proliferator-activated receptor gamma (PPARγ) binding activities and insulin-sensitizing effects. The objective of this study was to investigate the association of different ARBs with new-onset diabetes mellitus.

**Methods:**

In the respective cohort, a total of 492,530 subjects who initiated ARB treatment between January 2004 and December 2009 were identified from Taiwan National Health Insurance Database. The primary outcome was newly diagnosed diabetes, defined as at least one hospital admission or two or more outpatient visits within a year with an ICD-9-CM code 250. Cox proportional regression was used to estimate the risk of diabetes associated with each ARB, using losartan as the reference.

**Results:**

A total of 65,358 incident diabetes cases were identified out of 1,771,173 person-years. Olmesartan initiators had a small but significantly increased risk of developing diabetes after adjusting for baseline characteristics and mean daily dose (hazard ratio [HR], 1.07; 95% confidence interval [CI], 1.03-1.12). After excluding those followed for less than one year, the increase in diabetes risk are more pronounced (HR, 1.09; 95% CI, 1.05-1.14). This association was consistent across all subgroup analyses. Similar results were observed when a more strict definition of diabetes combining both diabetes diagnosis and anti-diabetic treatment was used. On the other hand, there was no difference in diabetes risk between telmisartan and losartan.

**Conclusions:**

Among all ARBs, olmesartan might be associated with a slightly increased risk of diabetes mellitus. Our data suggest differential diabetes risks associated with ARBs beyond a class effect.

## Introduction

Angiotensin II type 1 receptor blockers (ARBs) are widely used for treatment of hypertension and congestive heart failure. Several meta-analyses, randomised clinical trials or retrospective studies have demonstrated that ARBs use reduces diabetes risk in patients with hypertension or congestive heart failure as compared to other antihypertensive therapies or placebo [1-16]. Since hypertension is often associated with insulin resistance and impaired glucose tolerance, the metabolic effect of anti-hypertensive agents is viewed as an important consideration for choosing initial therapy. Accordingly, the UK National Institute for Health and Clinical Excellence now recommends ACE inhibitors and ARBs as the first-line antihypertensive drugs treatment partly because of their beneficial metabolic effects [4].

The anti-diabetic action of ARBs appears to be complex, including activation of peroxisome proliferator-activated receptor-γ (PPARγ), suppression of oxidative stress, inhibition of fibrosis, and enhancement of insulin signalling [17,18]. There is substantial difference in the chemical structure and lipid solubility among ARBs [19]. Furthermore, different ARBs had different degrees of PPARγ agonist activities. Telmisartan has highest PPARγ agonist activity, followed by candesartan and irbesartan; whereas losartan, valsartan and olmesartan seem to possess little PPARγ agonist activity [20-23]. The heterogeneity among ARBs might affect their metabolic action. Since there is currently no study comparing the risk of diabetes associated with individual ARBs, the objective of this study was to assess the association of individual ARBs with new-onset diabetes.

## Methods

### Data source

A single-payer and compulsory National Health Insurance (NHI) program was implemented in Taiwan since 1995. The enrollment rate was 99% in 2010. The Taiwan NHI database includes complete outpatient visits, hospital admissions, prescriptions, disease and vital status for 99% of total Taiwan population (approximately 23 million). Several computerized claims datasets were linked with the National Death Registry through the use of birth dates and civil identification numbers unique to each beneficiary. The protocol of this study was approved by the National Taiwan University Hospital Research Ethics Committee.

### Study population

From the source population, we identified adult patients aged more than 20 years who initiated losartan, valsartan, irbesartan, candesartan, telmisartan, or olmesartan treatment (anatomical therapeutic chemical [ATC] classification system codes were provided in Additional file 1: Table S1) between January 1, 2004 (when the above ARBs were all reimbursed by NHI) and December 31, 2009. Initiation was defined as free of any prescription of ARBs or ACE inhibitors 12 months prior to the first prescription (index date). Exclusion criteria were the patients 1) aged more than 100 years, 2) who did not have continuous NHI coverage 12 months before the index date, 3) who received more than one ARBs or ACE inhibitors on the index date, 4) who had diabetes diagnosis or received anti-diabetic therapy (insulin or oral anti-diabetic agents) before the index date, and 5) who already had cancer diagnosis before the index date.

### Use of study drugs

We collected information of prescribed drug types, dosage, date of prescription, supply days, and total number of pills dispensed from the pharmacy prescription database. Every person-day during study period was classified into current use and nonuse. Current use was defined as use during the period between the prescription start date and the end of the days of supply. Cumulative dosage of ARBs during the study period was calculated and presented as the defined daily dose (DDD), which was established by an expert panel according to the relative amount compared to the typical maintenance dose for an adult (DDDs: 50 mg for losartan, 80 mg for valsartan, 150 mg for irbesartan, 8 mg for candesartan, and 40 mg for telmisartan) [24]. Subsequently, the average daily dose for each individual was calculated by dividing the cumulative dosage by the follow-up duration.

### Outcome ascertainment

The primary outcome was diabetes incidence. Patients were classified as having newly diagnosed diabetes if they had at least one hospital admission with a diagnostic code of diabetes (The International Classification of Diseases, 9th Revision, Clinical Modification, ICD-9-CM code 250) or two or more outpatient visits with diabetic diagnostic code within a year. This definition of diabetes was evaluated by a study sampling 9,000 patients with a diagnosis of diabetes in the NHI claims data in 2000. Validation of this algorithm demonstrated a high level of sensitivity and positive predictive value (93.2% and 92.3%, respectively) [24].

### Covariate ascertainment and adjustment

We used inpatient and outpatient diagnosis files and prescription file during the 12-month period before the index date to ascertain patients’ history of hypertension, cardiovascular, cerebrovascular, peripheral vascular disease, chronic kidney, liver, and lung disease, and depression (ICD-9-CM codes provided in Additional file 1: Table S1) as well as the use of anti-platelet agents, anticoagulants, alpha-blockers, beta-blockers, calcium channel blockers, diuretics, other anti-hypertensive agents, nitrates, statins, fibrates, digitalis, anti-arrhythmic agents,cyclooxygenase-2 selective and non-selective non-steroidal anti-inflammatory drugs (NSAID) (ATC codes provided in Additional file 1: Table S1). We also collected patients’ information on age, sex, and patients’ resource utilization (number of outpatient visits, number of hospitalizations, number of laboratory test measurements) 12 months prior to the index date.

### Statistical analysis

Baseline characteristics, co-morbidities, medication use, and resource utilization among individual ARB initiators were presented. We computed their person-days of follow-up in each ARB use category. The crude incidence rates of diabetes and their 95% confidence intervals (CIs) were estimated based on a Poisson distribution. In the main analysis, we followed all ARBs users until the new onset of DM, death, disenrollment from the national health insurance, or study end (last outpatient visit or hospitalization before December 31, 2010). This intention-to-treat (ITT) analysis, analogous to ITT approach in randomized controlled trials, was based on the initial treatment assignment and not on the treatment eventually received. No matter how the medication had changed, all of the follow-up was assigned to belong to the initial ARB treatment group [25,26]. Cox proportional hazards regression model was used to calculate the hazard ratios [27] and their 95% CI with losartan as the common reference group. In the multivariable analysis, stepwise selection was used to control for variables with *p* values < 0.10 for model entry and > 0.05 for removal. In addition, time-varying covariate for mean daily dosage of ARB use was also adjusted in the multivariable regression model to control for the potential effect of dosage.

In the sensitivity analyses, we investigated whether effect estimates would change in response to more strict definition of outcome (which combined both diabetes diagnosis and anti-diabetic treatment) and exclusion of patients that were followed less than one year. Additionally, stratified analyses were performed to evaluate potential effect modification. Participants were further stratified according to 1) age (< 70, ≥ 70 years), 2) gender (men, women), and 3) whether having hypertension at the beginning. A test of interaction was performed using likelihood ratio test. Two-sided *p* value < 0.05 was considered to be statistically significant. All statistical analyses were performed with SAS 9.2 (SAS Institute, Cary, NC).

Auxiliary analyses were then conducted to compare the associations between ARB use and new onset DM among exclusive users who remained on the initial treatment throughout the follow-up course. This was used to examine whether the results changed substantially in comparison to the main ITT approach, as a measure of internal consistency.

## Results

**Figure 1:**
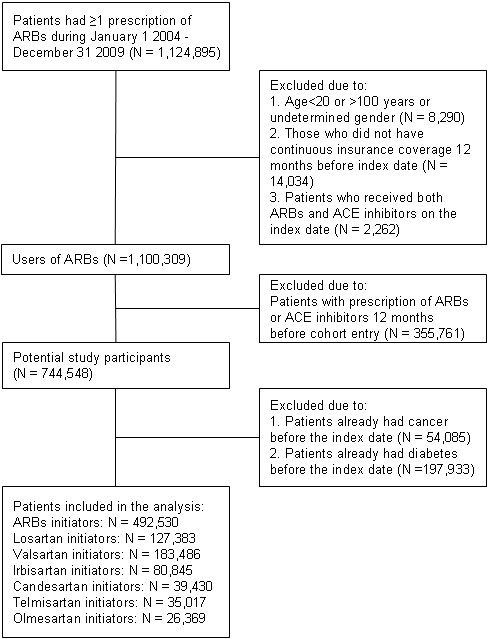
Study flow diagram.

A total of 492,530 ARBs initiators fulfilling the criteria were included in the analysis (Figure 1). The baseline characteristics for each ARB initiator is listed in Table 1. As shown in Table 1, groups of ARBs initiators differed in a number of baseline characteristics. Telmisartan and olmesartan initiators had a higher proportion of hypertension, but lower proportion of ischemic heart disease and heart failure, while olmesartan initiators were more likely to receive calcium channel blockers but less likely to receive anti-platelet and beta-blockers therapy. In contrast, candesartan initiators had a higher proportion of ischemic heart disease and heart failure, and were more likely to receive anti-platelet agents, beta-blockers, nitrates, and statins. Meanwhile, higher proportion of valsartan and irbesartan initiators had cerebrovascular disease and ischemic stroke.

**Table 1:** Baseline characteristics, comorbidities, medication use, and resource utilization 12 months before study entry among initiators of different angiotensin receptor blockers

	**Losartan**	**Valsartan**	**Irbesartan**	**Candesartan**	**Telmisartan**	**Olmesartan**
Number of patients	127,383	183,486	80,845	39,430	35,017	26,369
*Patient characteristics*						
Age at ARBs initiation (mean ± SD)	59.55 ± 14.39	60.00 ± 14.41	59.20 ± 14.39	59.67 ± 14.35	58.82 ± 14.12	58.45 ± 14.28
Male (%)	52.64	52.88	54.12	52.88	52.67	54.96
Initiation year (%)						
2004	21.16	23.23	25.33	7.22	21.06	0.88
2005	14.87	18.52	18.68	10.06	15.53	9.15
2006	11.54	15.59	15.97	17.58	13.72	14.77
2007	12.52	14.84	14.27	21.41	16.91	20.40
2008	19.21	13.60	13.42	22.74	16.57	27.46
2009	20.71	14.23	12.33	20.99	16.20	27.34
*Comorbidities (%)*						
Hypertension	87.05	88.36	88.68	85.87	90.72	91.20
Ischemic heart disease	19.19	20.78	19.80	27.30	19.76	18.22
Myocardial infarction	0.91	1.03	0.90	1.24	0.76	0.53
Heart failure	5.31	6.21	5.15	8.34	3.99	3.30
Atrial fibrillation	2.37	2.19	2.18	3.06	1.35	1.21
Cerebrovascular disease	11.01	14.17	14.68	11.32	11.91	11.08
Ischemic stroke	6.08	7.72	8.06	5.91	5.88	5.74
Intracerebral hemorrhage	1.13	2.30	2.16	1.36	1.53	1.39
Peripheral vascular disease	0.05	0.04	0.03	0.03	0.06	0.04
Chronic renal failure	3.48	3.15	4.28	2.10	2.51	2.24
Chronic liver disease	10.62	10.37	10.55	10.99	10.42	10.12
Chronic lung disease	17.12	18.04	16.80	17.22	16.22	16.43
Depression	4.08	4.28	4.30	4.18	4.18	4.00
Charlson’s index (mean ± SD)	0.78 ± 1.03	0.83 ± 1.05	0.84 ± 1.06	0.78 ± 1.01	0.73 ± 0.99	0.68 ± 0.95
Number of different ICD-9 diagnoses (mean ± SD)	13.62 ± 7.69	13.74 ± 7.74	13.37 ± 7.57	13.63 ± 7.70	13.21 ± 7.54	13.21 ± 7.59
Number of cardiovascular-related diagnoses (mean ± SD)	1.60 ± 1.10	1.71 ± 1.15	1.70 ± 1.15	1.79 ± 1.15	1.66 ± 1.07	1.59 ± 1.01
*Medication use (%)*						
Aspirin	28.39	31.83	29.27	31.48	27.88	27.62
Clopidogrel	2.32	2.90	3.12	3.83	2.28	1.85
Warfarin	1.36	1.33	1.25	1.66	0.92	0.86
Alpha-blockers	4.99	5.04	5.50	4.30	5.24	4.97
Beta-blockers	40.82	43.54	45.64	47.42	44.46	41.56
Calcium channel blockers	56.13	59.86	61.55	60.50	60.12	62.00
Diuretics	26.63	27.82	27.89	26.65	24.85	23.47
Other anti-hypertensive agents	1.54	1.84	2.04	1.48	1.68	1.56
Nitrates	10.87	12.83	11.68	15.74	10.91	9.79
Statins	12.02	11.81	13.64	15.55	13.16	12.21
Fibrates	4.17	4.22	4.54	4.45	4.70	4.08
Digitalis glycoside	3.15	3.40	2.64	3.45	2.08	1.72
Antiarrhythmics class I and III	3.30	3.33	3.35	4.15	2.52	2.43
COX-2 non-selective NSAIDs	76.74	75.97	74.47	73.31	74.47	75.58
COX-2 selective NSAIDs	7.49	8.24	8.47	7.32	7.12	5.94
Number of different prescription drugs (mean ± SD)	24.46 ± 15.72	25.41 ± 16.34	24.48 ± 15.93	23.69 ± 15.46	23.62 ± 15.61	23.48 ± 15.32
Number of cardiovascular-related medications (mean ± SD)	3.53 ± 2.20	3.76 ± 2.32	3.79 ± 2.31	3.83 ± 2.32	3.65 ± 2.23	3.58 ± 2.20
*Resource utilization (mean ± SD)*						
Number of A_1_C measurement	0.04 ± 0.20	0.04 ± 0.19	0.05 ± 0.21	0.06 ± 0.24	0.05 ± 0.21	0.04 ± 0.21
Number of lipid-related lab test	1.12 ± 1.47	1.20 ± 1.48	1.42 ± 1.53	1.68 ± 1.59	1.47 ± 1.56	1.37 ± 1.57
Number of cardiac ultrasound examination	0.15 ± 0.42	0.17 ± 0.46	0.18 ± 0.49	0.34 ± 0.65	0.19 ± 0.47	0.15 ± 0.44
Number of outpatient visits	25.62 ± 20.22	25.40 ± 20.37	24.96 ± 19.83	25.21 ± 19.99	24.32 ± 19.51	24.38 ± 20.08
Number of emergency department visit	0.41 ± 1.01	0.49 ± 1.20	0.47 ± 1.09	0.48 ± 1.21	0.42 ± 1.00	0.43 ± 0.97
Number of cardiology outpatient visits	1.59 ± 3.67	1.73 ± 3.70	2.15 ± 4.08	3.01 ± 4.62	2.09 ± 3.96	1.69 ± 3.57
Number of cardiovascular-related physician visits	5.82 ± 6.75	6.08 ± 6.98	6.23 ± 6.85	6.35 ± 6.88	5.98 ± 6.84	5.77 ± 6.68
Coronary revascularization %	0.44	0.57	0.49	1.03	0.39	0.49
Number of hospitalizations	0.23 ± 0.63	0.29 ± 0.69	0.28 ± 0.66	0.27 ± 0.64	0.22 ± 0.61	0.25 ± 0.65
Number of hospitalizations due to cardiovascular-related diseases	0.13 ± 0.43	0.17 ± 0.48	0.16 ± 0.47	0.16 ± 0.45	0.13 ± 0.43	0.14 ± 0.44
Number of hospital days	2.47 ± 16.01	3.01 ± 16.33	2.79 ± 14.54	2.46 ± 13.96	2.15 ± 13.53	2.57 ± 17.20
Number of cardiovascular-related hospital days	1.23 ± 8.39	1.69 ± 9.61	1.61 ± 8.82	1.34 ± 6.89	1.17 ± 7.32	1.35 ± 9.37

ARB: angiotensin receptor blockers.

The mean follow-up duration was 3.59 years. During a total of 1,771,173 person-years of follow-up, 65,358 incident diabetes cases were identified. The crude incidence rates of diabetes for different groups of ARB initiators are shown in Table 2. The crude HR of diabetes was higher for olmesartan initiators as compared with losartan initiators (HR, 1.35; 95% CI: 1.29-1.40, Table 3). After adjusting for differences in baseline characteristics, diabetes risk associated with olmesartan initiators remained significantly higher (adjusted HR, 1.07; 95% CI: 1.03-1.12, Table 3). The results were similar after further adjustment for mean daily dose (Table 3). After excluding those followed for less than one year, the crude and adjusted HR associated with olmesartan initiators increased to 1.48 (95% CI: 1.41-1.55) and 1.09 (95% CI: 1.05-1.14), respectively (Table 3). However, other ARBs including telmisartan, which has been shown to possess potent PPARγ activity were not associated with altered diabetes risk.

**Table 2:** Person-days and crude incidence rates of diabetes among initiators of angiotensin receptor blockers

	**Losartan**	**Valsartan**	**Irbesartan**	**Candesartan**	**Telmisartan**	**Olmesartan**
Exposed person-days	160,956,721	252,964,999	115,243,240	44,349,122	46,800,437	26,163,756
Mean follow-up days	1263.57	1378.66	1425.48	1124.76	1336.51	992.22
Mean treatment duration (days)	312.03	349.32	379.50	344.67	329.76	271.00
Mean daily dosage (DDD)	0.36	0.39	0.39	0.41	0.42	0.37
Number of newly diagnosed diabetes	16,227	25,849	11,436	4,371	4,666	2,809
Crude incidence rate per 1,000,000 person-days (95% CI)	100.82 (99.26-102.37)	102.18 (100.94-103.43)	99.23 (97.41-101.05)	98.56 (95.64-101.48)	99.70 (96.84-102.56)	107.36 (103.39-111.33)

**Table 3:** Hazard ratios of diabetes incidence comparing individual angiotensin receptor blocker with losartan

	**Valsartan**	**Irbesartan**	**Candesartan**	**Telmisartan**	**Olmesartan**
**All subjects**					
Crude	0.99 (0.97-1.01)	0.95 (0.93-0.97)	1.13 (1.09-1.17)	0.98 (0.95-1.01)	1.35 (1.29-1.40)
Multivariable regression analysis	1.01 (0.99-1.03)	0.99 (0.97-1.02)	0.99 (0.96-1.03)	0.99 (0.96-1.03)	1.07 (1.03-1.12)
Multivariable regression adjusted for mean daily dosage	1.01 (0.99-1.03)	0.99 (0.97-1.01)	0.99 (0.96-1.02)	0.99 (0.96-1.02)	1.07 (1.03-1.12)
**Excluding those followed for less than one year**					
Crude	1.00 (0.98-1.02)	0.97 (0.94-0.99)	1.18 (1.14-1.23)	1.00 (0.96-1.03)	1.48 (1.41-1.55)
Multivariable regression analysis	1.02 (1.00-1.04)	1.01 (0.98-1.03)	1.00 (0.96-1.04)	1.00 (0.96-1.03)	1.09 (1.04-1.14)
Multivariable regression adjusted for mean daily dosage	1.02 (1.00-1.04)	1.00 (0.98-1.03)	0.99 (0.96-1.03)	1.00 (0.96-1.03)	1.09 (1.05-1.14)

Multivariable regression analysis: adjusted for baseline covariates by traditional multivariable regression with stepwise variable selection.

In the stratified analysis, we found that the increased HR of diabetes associated with olmesartan initiators was consistent across all subgroups (Table 4). In sensitivity analysis using strict diabetes definition (diagnosis code plus anti-diabetic treatment), olmesartan was still associated with an increased risk (Additional file 1: Table S2). Furthermore, a decreased diabetes risk was also found for candesartan initiator using this strict diabetes definition (HR, 0.91; 95% CI: 0.87-0.95, Additional file 1: Table S2).

**Table 4:** Adjusted hazard ratios of diabetes incidence comparing individual angiotensin receptor blocker with losartan among different subgroups

	**Valsartan**	**Irbesartan**	**Candesartan**	**Telmisartan**	**Olmesartan**
Age < 70 years	1.01 (0.99-1.04)	1.01 (0.98-1.04)	1.02 (0.98-1.06)	1.01 (0.97-1.05)	1.07 (1.02-1.12)
Age ≧ 70 years	1.00 (0.96-1.04)	0.95 (0.91-0.99)	0.92 (0.86-0.99)	0.95 (0.89-1.01)	1.09 (1.01-1.18)
Men	0.99 (0.97-1.02)	0.98 (0.95-1.01)	1.00 (0.95-1.05)	0.98 (0.94-1.03)	1.08 (1.02-1.14)
Women	1.03 (1.00-1.06)	1.01 (0.97-1.04)	0.99 (0.94-1.04)	1.00 (0.96-1.05)	1.06 (1.00-1.13)
With hypertension	1.01 (0.99-1.03)	0.98 (0.96-1.01)	0.99 (0.95-1.02)	0.98 (0.95-1.02)	1.07 (1.02-1.11)
Without hypertension	1.02 (0.96-1.08)	1.07 (0.99-1.15)	1.03 (0.93-1.14)	1.09 (0.97-1.22)	1.13 (0.98-1.31)

The auxiliary analyses, which included only exclusive users who maintained the initial ARB throughout the follow-up period, showed similar results as compared to the main ITT approach. For example, the new DM risk was also slightly increased in olmesartan than in losartan (Additional file 1: Table S3).

## Discussion

Clear evidence indicated that the protective effects of ARBs against progression to diabetes were superior to those of placebo or other active anti-hypertensive treatment [1-16]. Meta-analyses showed that, overall, ARBs were associated with a 15 ~ 25% reduction in the risk of diabetes [3,28]. However, no solid evidence shows that all ARBs pose the same protective effect against diabetes. Instead, our findings indicate differential associations of ARBs with diabetes risk. In a large nationwide cohort, we found that olmesartan posed a slightly higher diabetes risk while candesartan seem to be associated with reduced diabetes risk.

Our data was in part in line with previous studies showing consistent protective effects of candesartan on diabetes risk. In comparison with placebo, candesartan therapy was associated with fewer newly diagnosed diabetes in patients with heart failure [5] and possibly also in the elderly patients [6]. When compared with active treatment (i.e., amlodipine and non-ARB antihypertensive agents), candesartan was also associated with a lower risk of diabetes in hypertensive patients with or without coronary artery disease [10,11].

Previous studies demonstrated that ARBs possess various PPARγ activation activities. Telmisartan seems to have strongest activity, followed by candesartan and irbesartan; while other ARBs have little activities [20-23]. In our study, however, telmisartan was not associated with lower diabetes incidence as expected. This is consistent with a large randomized clinical trial showing no effect of telmisartan on diabetes incidence as compared to placebo [29]. The explanation for this discrepancy is not clear but may be due to pleiotropic effects of ARBs on glucose metabolism beyond PPARγ activation. Indeed, candesartan was shown to improve insulin sensitivity through PPARγ-independent mechanism and to increase insulin content in pancreatic beta-cells by attenuating oxidative stress [27,30]. Losartan, irbesartan and valsartan, and telmisartan have been shown to exert various anti-diabetic effects other than PPARγ activation, including augmentation of blood flow to muscle, direct modulation of insulin signaling and up-regulation of glucose transporter expression in muscle, reduction of islet fibrosis through inhibition of TGF-β, and activation of AMPK/SIRT1 pathway [31-35]. These data, together with our observation, support heterogeneous anti-diabetic effects of ARBs.

Our study has some unique strength. First, this is currently the only study comparing diabetes risk for individual ARB. Second, this study is a nationwide population-based cohort study. The huge sample size and long duration of follow-up enable this study adequately powered to detect difference among ARBs. Third, the intention-to-treat analysis preserves the baseline comparability of the treatment groups, reduces the potential bias due to drug switching or discontinuation, and provides a conservative estimate. Therefore, the results provided real-life estimations of diabetes risk associated with individual ARB, which have important clinical implications. Diabetes is a strong risk factor of cardiovascular disease, renal failure, and retinopathy which imposes enormous economic burden to the health care system [36-38]. The changed diabetes incidence with different ARB therapy would translate directly to mortality and medical costs. Fourth, different analytic strategies, including the ITT and exclusive user analyses, showed similar results and indicated an internal consistency among these results.

Our study has some limitations. First, this is an observational study but not a randomized clinical trial. Therefore, the association between individual ARBs and new-onset diabetes may be affected by unknown or unmeasured confounders. Laboratory and anthropometric data such as fasting glucose or body mass index were not available in the NHI database. However, these factors are unlikely to confound the results since physicians did not prescribe specific ARB according to these factors. Furthermore, our analyses have been fully adjusted for these baseline co-morbidities. Second, imperfect adherence to therapy or discontinuation of therapy could have attenuated our intention-to-treat effect estimate. Third, the definition of new-onset diabetes was made according to the ICD-9-CM code but not by screening tests. Although the definition algorithm using ICD-9-CM diagnosis code has a high positive predictive value, some diagnoses of diabetes might be missed. Nevertheless, the magnitude of increased risk associated with olmesartan changed little (from 7% to 5%) when more strict definition (diagnosis code plus anti-diabetic therapy) was applied in the sensitivity analysis. The under-estimation due to un-diagnosed diabetes or latent diabetes should be minimal.

In conclusion, we found a small but significantly increased diabetes incidence in olmesartan initiators as compared to losartan initiators in a large nationwide population-based cohort. Telmisartan was not associated with reduced diabetes incidence. These findings suggest heterogeneous diabetes risks associated with different ARBs beyond a class effect, but the difference in diabetes risk does not seem to correlate with PPARγ activation activities. Further study is needed affirm this observation.

## Consents

This study was approved by the Institutional Reviewer Board of National Taiwan University Hospital. Due to the anonymous data analyses, written informed consents were waived.

## Abbreviations

ARB: Angiotension II type 1 receptor blockers; ACE: Angiotensin-converting enzyme; PPARγ: Peroxisome proliferator-activated receptor – gamma; NHI: National health insurance; ATC: Anatomical therapeutic chemical; DDD: Defined daily dose; ICD-9-CM: International classification of diseases, 9th revision, clinical modification; CI: Confidence intervals; HR: Hazard ratio.

## Competing interests

All authors declared no competing interests.

## Authors’ contributions

Authors Contribution: study concept and design: CHC, JWL; acquisition of data: MSL; analysis and interpretation of data: CHC, YCC, JWL; drafting of the manuscript: YCC, JWL, CHC; critical revision of the manuscript for important intellectual content: YCC, MSL, LMC; statistical analysis: LCW; study supervision: JWL, LMC, MSL. All authors read and approved the final manuscript.

## Supplementary Material

Additional file 1: Table S1 ICD-9-CM codes and ATC codes used in this study. **Table S2** Hazard ratios of diabetes incidence comparing users of individual angiotensin receptor blocker with losartan after excluding those followed for less than one year. **Table S3** Hazard ratios of diabetes incidence comparing exclusive users of individual angiotensin receptor blocker with losartan. Click here for file
