# Supplementary material for: Different angiotensin receptor blockers and incidence of diabetes: a nationwide population-based cohort study
Source: Cardiovasc Diabetol. 2014 May 14;13:91. doi: 10.1186/1475-2840-13-91 (PMC4039330; doi:10.1186/1475-2840-13-91)
Supplement: Additional file 1: Table S1 — ICD-9-CM codes and ATC codes used in this study. Table S2 Hazard ratios of diabetes incidence comparing users of individual angiotensin receptor blocker with losartan after excluding those followed for less than one year. Table S3 Hazard ratios of diabetes incidence comparing exclusive users of individual angiotensin receptor blocker with losartan. [file 1475-2840-13-91-S1.docx]

**Supplementary Table 1.** ICD-9-CM codes and ATC codes used in this study

| **Comorbidities** | **ICD-9-CM codes** | **Medication** | **ATC codes** |
| --- | --- | --- | --- |
| Hypertension | 401-404 | Angiotensin receptor blockers | C09CA01, C09CA03, C09CA04, C09CA06, C09CA07, C09CA08, |
| Diabetes | 250 | ACE inhibitors | C09AA |
| Ischemic heart disease | 410-414 | Alpha-blockers | C02CA |
| Myocardial infarction | 410, 412 | Beta-blockers | C07A |
| Congestive heart failure | 428 | Calcium channel blockers | C08 |
| Atrial fibrillation | 427.31 | Diuretics | C03 |
| Cerebrovascular disease | 430-438 | Other anti-hypertensive agents | C02A, C02B, C02CC, C02D, |
| Ischemic stroke | 433, 434, 436 (exclude 800, 801, 802, 803, 804, 850, 851, 852, 853, 854 ,V57) | Nitrate | C01DA |
| Intracerebral hemorrhage | 430-432 | Insulin | A10A |
| Peripheral arterial disease | 440.2, 440.4, 443.81, 443.9 | Sulfonylurea | A10BB |
| Chronic renal failure | 403.01, 403.11, 403.91, 404.02, 404.03, 404.12, 404.13, 404.92, 404.93, 585 ,V45.1, V56.0, V56.8 | Metformin | A10BA02 |
| Chronic liver disease | 070.2x, 070.3x, V02.61, 070.41, 070.44, 070.51, 070.54, V02.62, 571.0, 571.1, 571.2, 571.3, 571.4, 571.5, 571.6 | Thiazolidinediones | A10BG02, A10BG03 |
| Chronic lung disease | 490-496, 500-508 | Glinides | A10BX02, A10BX03 |
| Depression | 296.2,296.3,298.0,300.4,309.0,309.1, 293.83,296.90,309.28,296.82, 311 | Alpha-glucosidase inhibitors | A10BF |
|  |  | DPP4 inhibitors | A10BH |
|  |  | Statins | C10AA |
|  |  | Fibrates | C10AB |
|  |  | Wafarin | B01AA03 |
|  |  | Aspirin | B01AC06, N02BA01 |
|  |  | Clopidogrel | B01AC04 |
|  |  | COX-2 nonselective NSAIDs | M01A (exclude M01AH, M01AX05) |
|  |  | COX-2 selective NSAIDs | M01AH |
|  |  | Digitalis | C01AA |
|  |  | Anti-arrhythmics Class I and III | C01B |

**Supplementary Table 2.** **Hazard ratios of diabetes incidence comparing users of individual angiotensin receptor blocker with losartan after excluding those followed for less than one year**

|  | Valsartan | Irbesartan | Candesartan | Telmisartan | Olmesartan |
| --- | --- | --- | --- | --- | --- |
| Crude | 1.00 (0.98-1.02) | 0.97 (0.94-0.99) | 1.18 (1.14-1.23) | 1.00 (0.96-1.03) | 1.48 (1.41-1.55) |
| Multivariable regression analysis | 1.02 (1.00-1.04) | 1.01 (0.98-1.03) | 1.00 (0.96-1.04) | 1.00 (0.96-1.03) | 1.09 (1.04-1.14) |
| Multivariable regression adjusted for mean daily dosage | 1.02 (1.00-1.04) | 1.00 (0.98-1.03) | 0.99 (0.96-1.03) | 1.00 (0.96-1.03) | 1.09 (1.05-1.14) |
| Sensitivity analysis | 0.99 (0.97-1.02) | 0.97 (0.94-1.00) | 0.92 (0.88-0.96) | 1.00 (0.96-1.05) | 1.06 (1.01-1.12) |

**Supplementary Table 3.** **Hazard ratios of diabetes incidence comparing exclusive users of individual angiotensin receptor blocker with losartan**

|  | Valsartan | Irbesartan | Candesartan | Telmisartan | Olmesartan |
| --- | --- | --- | --- | --- | --- |
| Crude | 0.99 (0.96-1.02) | 0.96 (0.93-1.00) | 1.11 (1.05-1.17) | 0.99 (0.93-1.04) | 1.29 (1.21-1.37) |
| Multivariable regression analysis | 1.03 (1.00-1.06) | 1.02 (0.98-1.06) | 0.97 (0.92-1.02) | 1.00 (0.95-1.06) | 1.05 (0.98-1.11) |
| Multivariable regression adjusted for mean daily dosage | 1.03 (1.00-1.06) | 1.02 (0.98-1.06) | 0.97 (0.92-1.02) | 1.00 (0.95-1.05) | 1.05 (0.99-1.12) |
| Sensitivity analysis^†^ | 0.97 (0.94-1.01) | 0.95 (0.91-1.00) | 0.87 (0.81-0.93) | 0.98 (0.91-1.05) | 1.06 (0.99-1.15) |

^†^Sensitivity analysis: diabetes outcome defined as with diagnostic codes and receiving anti-diabetic therapy
